# Mesoscopic Investigation of Conventional and Weakly Bonded Cement Stabilized Macadam Based on Discrete Element Method: Considering Realistic Particle Shape Effects

**DOI:** 10.3390/ma19122577

**Published:** 2026-06-15

**Authors:** Hao Zhang, Chunyu Liang, Yancong Zhang

**Affiliations:** 1 Shanxi Transportation Technology Research & Development Co., Ltd., Taiyuan 030032, China; 2 Transportation College, Jilin University, Changchun 130025, China

**Keywords:** cement stabilized macadam, discrete element method, unconfined compressive strength, realistic aggregate morphologies, mesoscopic analysis

## Abstract

Road engineers still face a critical challenge in improving the crack resistance of cement-stabilized macadam (CSM) base courses. This study employs the discrete element method (DEM) with realistic aggregate morphologies from X-ray computed tomography to model normally bonded and weakly bonded CSM. The mesoscopic parameters of normally bonded models were calibrated against laboratory unconfined compressive strength (UCS) tests, and a weakening ratio of bond strength (*Wr_bs_*) was introduced to define the weakly bonded model. The results show that UCS decreases monotonically with the reduction in *Wr_bs_* and the increase in *Rr_ca_*. The maximum strength reduction reaches 26.3% at the extreme condition of *Rr_ca_* = 100% and *Wr_bs_* = 50%. Despite this reduction, the UCS of weakly bonded specimens remains compliant with Chinese specifications for base course materials when designed with appropriate parameters. Notably, weakly bonded specimens exhibit a more dispersed crack distribution and a more gradual energy dissipation process. This mechanism is associated with a reduced tendency for macroscopic crack initiation and propagation, suggesting the potential of weakly bonded CSM to enhance crack resistance. This work provides a mesoscopic theoretical foundation for the engineering application and sustainable development of weakly bonded CSM in pavement base courses.

## 1. Introduction

Semi-rigid base asphalt pavement is the primary pavement structure in China [1,2]. The most common semi-rigid base is the cement stabilized macadam (CSM) base, which has good bearing capacity and plate-forming property [3,4]. However, CSM is highly prone to the formation of drying shrinkage cracks and temperature shrinkage cracks triggered by fluctuations in temperature and humidity [5,6]. These cracks will gradually propagate into reflective cracks, thereby severely compromising the service life of asphalt pavements [7,8,9]. The effective prevention and control of reflection cracks has long been a research hot spot in the field of road engineering.

In previous practices, the primary measures to solve the cracking problem of CSM are as follows:

(1) Optimizing the mixture proportion involves reducing the content of particles smaller than 0.075 mm, controlling the cement dosage, and adopting a skeleton structure [10,11]. Studies indicate that optimized gradation design can effectively reduce the drying shrinkage coefficient by 11.9% and the temperature shrinkage coefficient by 14.4% [12].
(2) Enhancing the crack resistance of the semi-rigid base by incorporating fiber materials into CSM [13,14]. Existing research indicates that incorporating 1% plant fiber with a length of 15 mm into CSM can reduce the drying shrinkage coefficient of the material by 29% [13].
(3) Adding expansive agents to CSM to offset shrinkage strain [15]. Previous studies show that the maximum reduction in drying shrinkage coefficient reaches 18.92% by adding expansive admixture.
(4) Releasing stress by making cutting joints on the base course or generating micro-cracks through compaction. Long-term field observations reveal that for pavement sections with a 4% cement content, microcracking technology reduces cracking by nearly 50% compared with conventional moist curing [16].

Currently, the cracking problem of CSM can be alleviated by optimizing the mixture proportion, incorporating fiber materials, and blending with additives. However, these measures still suffered from the problems of high construction difficulty and elevated costs.

In recent years, micro-cracking technology has been introduced into the research on crack prevention and the control of CSM. Micro-cracking technique entails the deliberate introduction of micro-cracks during production. It mitigates damage caused by the formation of macro-cracks by regulating the initiation and propagation of these micro-cracks. The micro-cracking technique is categorized into two methodologies: the compaction-induced micro-cracking technique and the internally induced micro-cracking technique.

The compaction-induced micro-cracking technique employs secondary vibratory rolling to generate micro-cracks within the cement-stabilized macadam (CSM) base, thereby reducing the adverse shrinkage stress induced by cement hydration. This technique was initially proposed by Litzka and Lehner [17] and implemented in practical engineering applications. Field testing indicated that micro-cracking technology can effectively mitigate the formation of pavement reflective cracks. Mitiche-Kettab et al. [18] confirmed that the micro-cracks induced by the micro-cracking technique in CSM are controllable and conducted a comprehensive mechanical analysis using finite element software. Brandl [19] demonstrated in his research that the adoption of micro-cracking technology significantly mitigated pavement reflective cracks in the Austria–Hungary highway project. This conclusion has also been validated by the research of Tom and Stephen [20] from the United States. And this technique has been successfully applied to the quarter CSM base in Texas.

The induced micro-cracking technique weakens interface bond strength between coarse aggregate and cement mortar through the surface coating of coarse aggregate. This promotes preferential crack initiation at the interfaces to randomize and disperse damage within the matrix.

In practice, the weakly bonded interface is typically created by coating the coarse aggregate with a flexible material (e.g., asphalt emulsion), which reduces the bond strength between the cement mortar and the aggregate. Lei Quan et al. [21] weakened the interface bonding strength through the micro-cracking agent. The experimental results indicated that increasing the replacement ratio of weakly bonded aggregate reduced the mechanical properties but enhanced the shrinkage and frost resistance of the cement-stabilized material. Jian Sun et al. [22] observed consistent trends: incorporating emulsified asphalt decreased the UCS of CSM while improving its flexural tensile strength and frost resistance. Jinbo Liu [23] reported that weakly bonded CSM showed reductions in the dry shrinkage coefficient, temperature shrinkage coefficient, and water loss rate, which correspond to its superior shrinkage resistance. To validate its crack resistance, Yuan Zhao [24] constructed a test road using weakly bonded CSM. The field data showed that applying a weakening agent at 10% of the cement content effectively reduced macro-cracking in the semi-rigid base. Shaowen Du [25] evaluated CSM incorporating emulsified asphalt and found that it reduced the UCS while enhancing dry shrinkage resistance.

However, these studies mainly focus on the changes in macroscopic properties induced by weak bonding, whereas the material’s strength attenuation law, mesoscopic damage mechanism, and energy evolution mechanism remain unclear. In this paper, numerical simulation models for normal bonding and weakly bonded CSM were established using the discrete element method (DEM). The reduction in UCS of weakly bonded CSM was quantified by varying the replacement ratio of coarse aggregate and the weakening ratio of bond strength. Furthermore, the energy dissipation mechanism during loading and crack evolution behavior were elucidated. This study provides a theoretical foundation for the development and application of weakly bonded CSM.

## 2. Materials and Methods

### 2.1. Materials and Gradations

Jidong brand Grade 425 Ordinary Portland Cement (OPC) was used in this study, with a fixed dosage of 5% by mass of aggregates. Its technical properties are summarized in Table 1.

The aggregate was limestone sourced from LouFan quarry. The technical indexes of the aggregate were tested in accordance with the China standard (JTG 3432, 2024) [26], as shown in Table 2 and Table 3.

The gradation of the CSM, designed in accordance with the standard (JTG-T-F20, 2015) [27], is present in Table 4.

### 2.2. Laboratory Test

**Figure 1:**
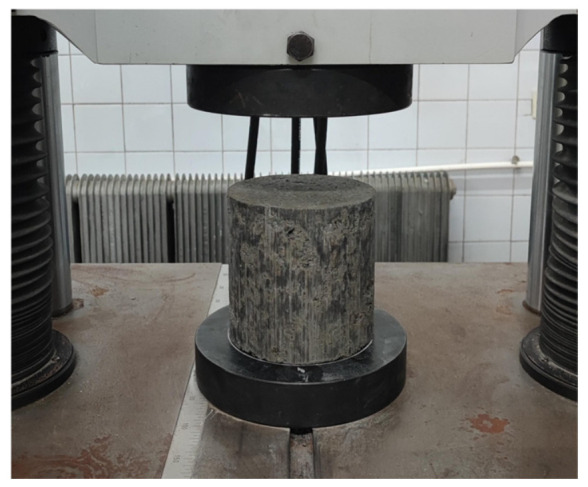
Unconfined compressive strength test.

Cylindrical specimens with dimensions of 150 mm × 150 mm were adopted for the unconfined compressive strength (UCS) test. All prepared samples were cured under controlled conditions of 20 °C ± 2 °C and relative humidity above 95%. Following a 7-day curing period, the UCS tests were conducted using a universal testing machine (as shown in Figure 1. The loading rate of the press was set to 1 mm/min, and the specimen was loaded at a constant speed until failure.

### 2.3. Numerical Modeling

#### 2.3.1. Realistic Aggregate Morphology

**Figure 2:**
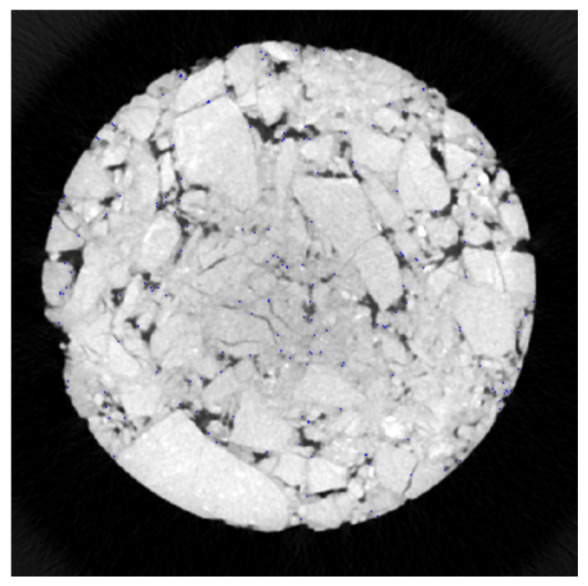
CT scanning image.

As a reliable non-destructive imaging technique, X-ray computed tomography (CT) enables the three-dimensional visualization of internal information within CSM specimens. In the present study, CT was employed to characterize the aggregate distribution within CSM specimens, as shown in Figure 2.

Aggregate morphology acquired from CT images via three-dimensional reconstruction in Avizo was imported into the DEM platform. Notably, reconstruction parameter configurations in Avizo govern the segmentation accuracy between aggregate and mortar, inevitably introducing morphological deviations to the extracted aggregate geometry. If experimental resources allow, individual aggregates can be scanned independently to obtain their true geometric profiles.

**Figure 3:**
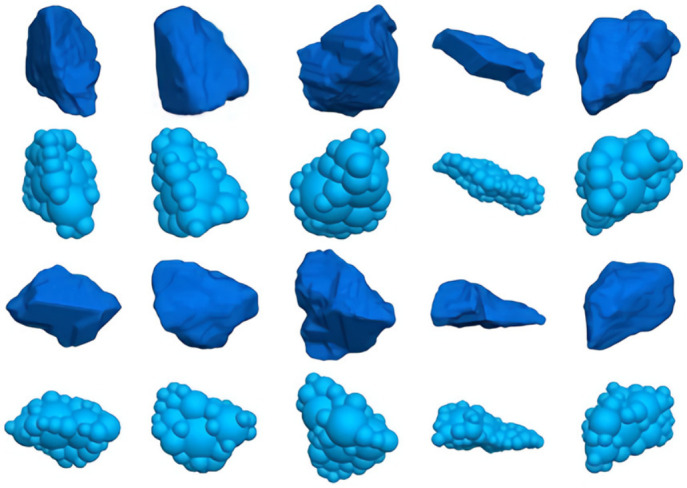
Characteristic clump templates.

Clump templates were generated using the ‘clump template create’ command. The fidelity of the templates was controlled by adjusting the ratio and distance parameters. Based on previous studies, the ratio and distance were set to 0.25 and 105, respectively. With this set of parameters, the aggregate morphology can be approximately captured without sacrificing computational efficiency; although it does not achieve a full geometric reconstruction of the original particles. The characteristic clump templates are shown in Figure 3.

#### 2.3.2. Numerical Modeling and Simulation Setup

The discrete element model was established based on the gradation of laboratory specimens and the cement content. In the model, coarse aggregate was modeled using realistic aggregate morphology, while fine aggregate and cement were represented by spherical particles. Considering both computational efficiency and simulation accuracy, the radius of the spherical particles was set to 1 mm.

**Figure 4:**
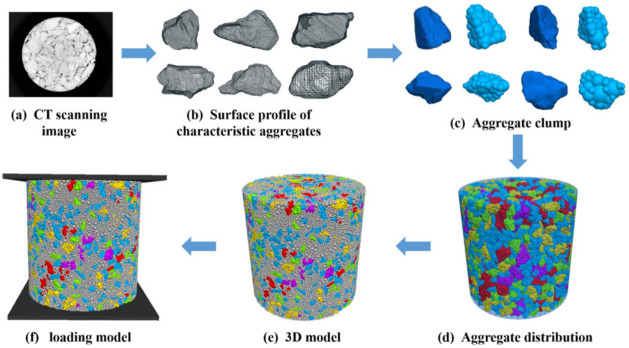
Numerical modeling process.

In the present study, aggregate was assumed to be unbreakable rigid bodies under applied loads. Aggregate was randomly generated and distributed within the CSM specimens, as illustrated in Figure 4d. The blue particles denote aggregate with a particle size ranging from 4.75 to 9.5 mm, while the green particles indicate those ranging from 9.5 to 13.2 mm. Meanwhile, the yellow particles represent those ranging from 13.2 to 16 mm, and the purple particles signify those ranging from 16 to 19 mm. In addition, red particles correspond to the largest particle group, with a size range of 19–26.5 mm. The numerical modeling process is shown in Figure 4. In total, the model was composed of 106,790 cement mortar particles and 2672 coarse aggregate particles within the size range of 4.75–26.5 mm.

In the discrete element method, two walls were generated at the top and bottom of the model to simulate the loading plates, as shown in Figure 4f. To balance simulation accuracy and computational efficiency of the simulation experiment, the loading velocity of the wall was set to 0.02 m/s.

##### Establishment of the Weakly Bonded CSM Model

Based on the normally bonded model, the numerical model of weakly bonded CSM was developed following the procedures below.

**Figure 5:**
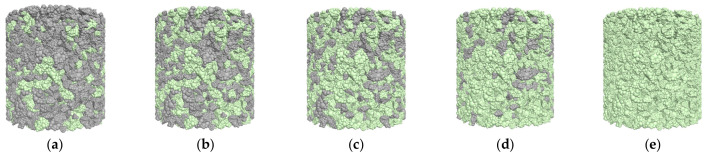
Specimens with different *Rr_ca_*: (**a**) 20%; (**b**) 40%; (**c**) 60%; (**d**) 80%; (**e**) 100%.

(1) The previously developed normally bonded model was imported into the discrete element method.
(2) Coarse aggregate at a specified proportion were randomly selected through programming, as shown in Figure 5.
(3) The interface bond strength between the selected coarse aggregate and cement mortar was weakened by a specified ratio, with the normal bond strength set as the reference.
(4) Finally, the loading plate was generated following the method described in the previous section.

#### 2.3.3. Contact Model

From a macroscopic perspective, the mechanical properties of CSM primarily arise from the cementation provided by cement and the skeletal support of the aggregate. In the discrete element model, both the cementation and skeletal support were characterized through contact models between particles. The contact model of the CSM mixture is shown in Table 5.

**Figure 6:**
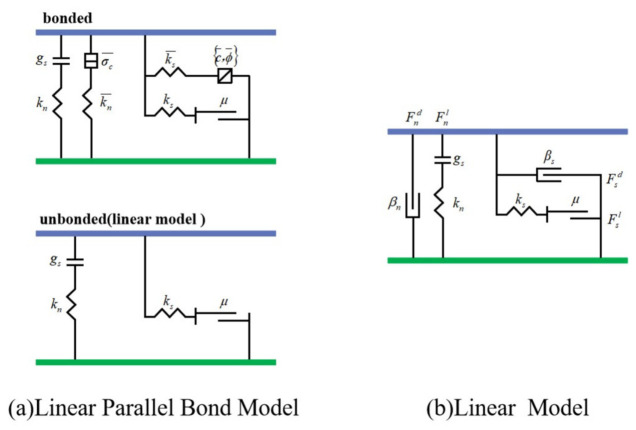
The schematic diagram of LPBM and Linear model.

The LPBM was appropriate for characterizing the mechanical behavior of bonded materials. Figure 6a illustrated the schematic diagram of the LPBM and Figure 6b illustrated the schematic diagram of the Linear model.

### 2.4. Determination of Parameters

#### 2.4.1. Normally Bonded Model

In discrete element simulations, the macroscopic mechanical behavior of the model is directly determined by the modeling accuracy and the mesoscopic parameters of inter-particle contacts. However, due to limitations in meso-scale measurement techniques, it remains extremely difficult to directly determine the mesoscopic parameters between particles in laboratory experiments. Therefore, most studies adopt parameter calibration methods to indirectly obtain the mesoscopic parameters. The core idea is to perform iterative adjustments of the mesoscopic contact parameters based on the results of the parameter sensitivity analysis of the contact model, by comparing the stress–strain curves from numerical simulations with those from laboratory tests. The calibration is considered successful when the error between the simulated and experimental stress–strain curves meets the acceptance criteria, indicating that the calibrated mesoscopic parameters can characterize the mechanical behavior of the material observed in the laboratory tests.

**Figure 7:**
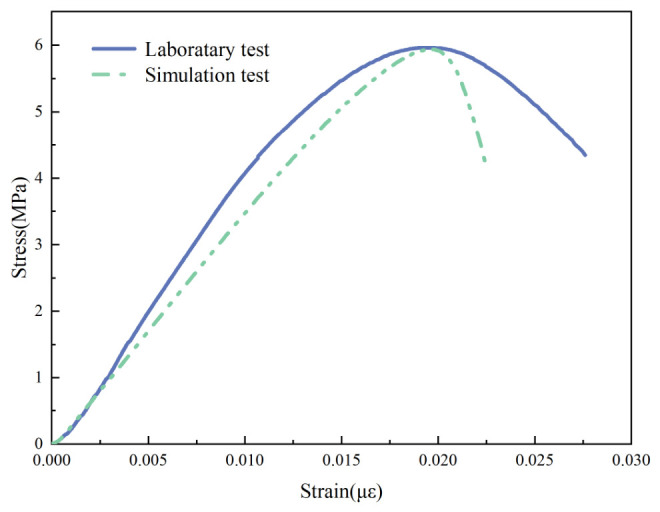
Stress–strain curve of UCS test.

On the basis of the parametric sensitivity analysis from previous investigations [28], the unconfined compressive strength test was used for parameter calibration, and the resulting comparison of stress–strain curves is illustrated in Figure 7.

The laboratory test yielded a peak stress of 5.96 MPa at a strain of 0.0196, while the simulation gave corresponding values of 5.93 MPa and 0.0192. The simulated curve closely resembles the laboratory test curve in terms of trend, slope, and peak value, with a stress error of only 0.5%.

**Figure 8:**
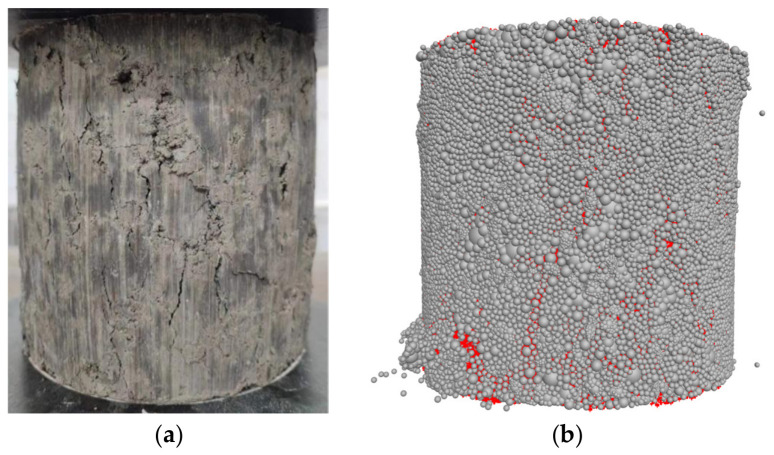
Comparison of fractures. (**a**) Crack propagation of specimen, (**b**) crack propagation in the simulation model.

The trend of the micro-crack expansion of the specimen is compared with that of the discrete elements model. As illustrated in Figure 8, both numerical and laboratory specimens develop reticular fracture patterns consisting of primary cracks and branched secondary cracks, with consistent failure morphologies and concentrated crack distribution in the middle region of the specimens. It is concluded that the established discrete element model can well reproduce the damage characteristics of laboratory-tested specimens. These findings demonstrate that the calibrated mesoscopic parameters (listed in Table 6) are capable of effectively characterizing the meso-scale mechanical behaviors of cement-stabilized macadam (CSM) within the discrete element framework.

#### 2.4.2. Weakly Bonded Model

In this study, the weakly bonded model was developed from the normally bonded model by degrading the bond parameters at the interface between cement mortar and coarse aggregate. The *Wr_bs_* was set to 90%, 80%, 70%, 60%, and 50%. The corresponding interface bonding parameters are presented in Table 7.

## 3. Numerical Simulation of Normally Bonded CSM

### 3.1. Failure Characteristics of Specimen

In laboratory tests, it is difficult to directly observe the internal failure behavior of CSM specimens. Therefore, acoustic emission (AE) technology is commonly employed to collect signals, which indirectly reflect the failure process [29].

**Figure 9:**
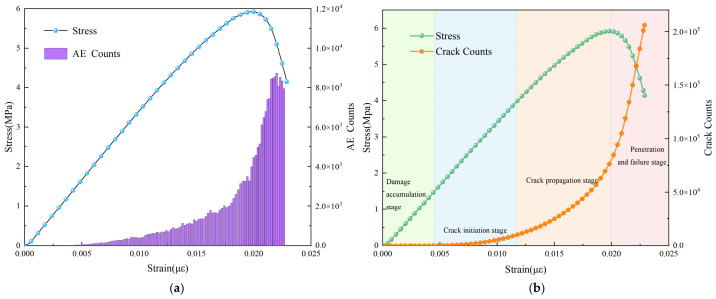
Micro-crack propagation during loading: (**a**) AE signal evolution; (**b**) evolution of failure stages for CSM.

AE technology is a non-destructive and dynamic detection technique widely used to monitor the initiation and propagation of micro-cracks [30,31,32,33]. The technique is based on the detection of transient elastic waves (acoustic emissions) generated by rapid energy release during material fracture. Sensors mounted on the specimen surface convert these mechanical waves into measurable electrical signals. In the DEM, AE signals were acquired through the secondary programming of the ‘fracture’ file. This study focuses on analyzing the synergistic variation characteristics of stress, cracks, and acoustic emission events based on strain growth, as illustrated in Figure 9.

The simulation results indicate that acoustic emission events were initiated in the model once the axial strain reached 4.52 × 10^−3^. With the increase in strain, the count of acoustic emission events increases continuously, and progressive failure occurs in the specimen. The failure process of CSM can be divided into four stages: damage accumulation stage, crack initiation stage, crack propagation stage, penetration, and failure stage. The features corresponding to each phase are described as follows:

Damage accumulation stage: No AE events were detected, while the specimen only underwent minimal elastic deformation as the load increased.

Crack initiation stage: A limited number of AE signals were initiated within the model, which indicated the initiation and gradual proliferation of micro-crack activity, with the inter-particle friction effect intensifying correspondingly.

Crack propagation stage: AE signals increased remarkably, which signifies that the existing micro-cracks have entered the stage of accelerated propagation, with the continuous initiation of new micro-cracks.

Penetration and failure stage: The count of AE events surged to a peak within a very limited strain interval. This demonstrated that micro-cracks rapidly accumulated, interconnected, and formed macroscopic main cracks during this phase. Subsequently, the main cracks traversed the entire specimen, leading to the loss of load-bearing capacity and a drastic drop in stress. Notably, the peak in acoustic emission activity occurs distinctly later than the peak stress.

### 3.2. Micro-Cracks Distribution

**Figure 10:**
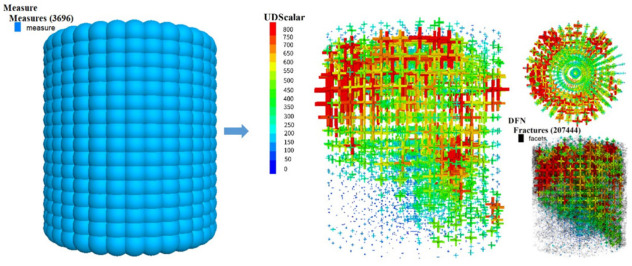
Spatial distribution cloud map of micro-cracks.

In the DEM, measurement spheres were employed to monitor local parameters in real time. To ensure uniform spatial coverage, 3696 regularly arranged spheres with a radius of 10.2 mm were generated. By programming with the Fish language, the number of micro-cracks within each measurement sphere was counted. Subsequently, a spatial distribution cloud map of micro-cracks was generated based on a color mapping scheme, as shown in Figure 10.

The cracks initiated and propagated in the CSM specimens under loading exhibited distinct heterogeneous distribution characteristics. These cracks predominantly concentrate in two zones. The first is the specimen top region, where micro-crack initiation preferentially occurs due to the stress concentration effect at the loading end. In this region, the crack distribution presents a “dense periphery and sparse center” pattern, which is mainly attributed to the initial structural defects within the specimen and the peripheral stress concentration induced by the end effect of the loading plate.

The second is the shear failure band oriented at approximately 45° to the axial loading direction; this band represents the preferential orientation for material shear failure. Within this zone, micro-cracks readily propagate and coalesce, ultimately forming a macroscopic main shear plane.

### 3.3. Evolution of Particle Displacement

**Figure 11:**
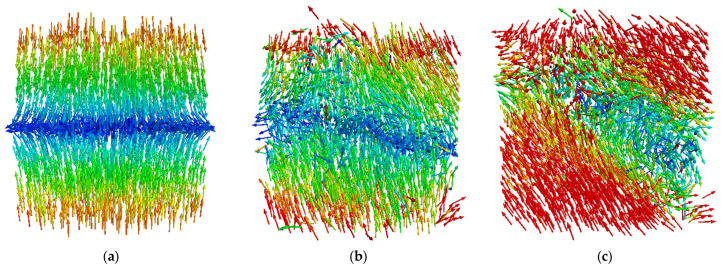
Particle velocity vectors during the UCS test: (**a**) Initial loading stage; (**b**) middle loading stage; (**c**) final loading stage.

The dynamic displacement behavior of aggregate underloading was successfully monitored. As illustrated in Figure 11, the displacement field exhibited a significantly non-uniform distribution pattern, which can be categorized into three distinct stages.

During the initial loading stage, the specimen primarily underwent axial compressive deformation. Most displacements were concentrated in the contact regions between the specimen and the loading plates, whereas a nearly horizontal low-displacement zone was formed in the middle of the specimen. Overall, the specimen’s displacement field remained relatively uniform at this stage.

In the middle loading stage, the horizontal displacement components of particles increased significantly, and the displacement vectors inclined outward. Consequently, the previously formed nearly horizontal low-displacement zone in the middle also tilted accordingly and lost its regular morphology. Correspondingly, the phenomenon of deformation localization began to emerge initially.

In the final loading stage, deformation became highly concentrated in local regions. The maximum displacement was focused on the diagonal areas of the lower left and upper right corners, with the displacement vectors displaying an obliquely symmetric distribution. Eventually, a through-going shear band with an inclination angle of approximately 45° was formed in the middle of the specimen.

### 3.4. Energy Evolution

**Figure 12:**
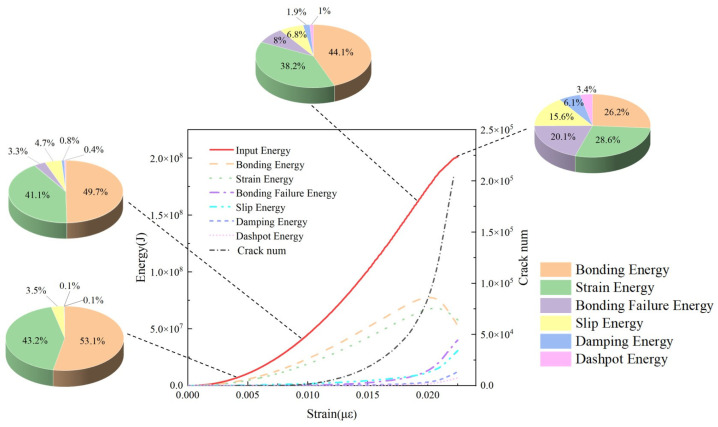
Energy evolution of UCS test. Bonding energy is elastic energy stored in pb-contact; Strain energy is elastic energy stored in contact; bonding failure energy is energy dissipated by bond failure; slip energy is energy dissipated by friction; damping energy is energy dissipated by damp; dashpot energy is energy dissipated by dashpots.

The energy within the system was monitored in real time throughout the loading process using the history command, and the corresponding results were illustrated in Figure 12. The energy primarily monitored comprised strain energy, bonding energy, and dissipated energy.

In the initial loading stage, the input energy was mainly converted into bonding energy (44.1%) and strain energy (52.0%). At this stage, the specimen only underwent elastic deformation, and no micro-cracks were initiated.

As the applied load increased, the relative movement between particles resulted in local stress exceeding the failure threshold, and micro-cracks began to initiate. This was manifested by the gradual increase in sliding friction energy and bonding failure energy.

With further loading, the existing micro-cracks continued to propagate accompanied by the initiation of new cracks. This caused a continuous increase in sliding friction energy and bonding failure energy, while the proportions of bonding energy and strain energy gradually decrease.

The material reached its ultimate load-bearing capacity when the strain energy and bonding energy peaked. Subsequently, with continuous loading, the stored strain energy and bonding energy were rapidly converted into dissipated forms such as slip energy, bonding failure energy, and damping energy. This triggered the sharp propagation of internal cracks within the specimen, driving the material into the main evolution stage of macroscopic failure. By the end of loading, the proportions of bonding energy and strain energy decreased to 26.2% and 28.6%, respectively. In summary, the essence of the macroscopic failure of CSM can be attributed to the energy conversion and dissipation-driven instability behavior.

## 4. Numerical Simulation of Weakly Bonded CSM

### 4.1. Mechanical Behavior of Weakly Bonded CSM

**Figure 13:**
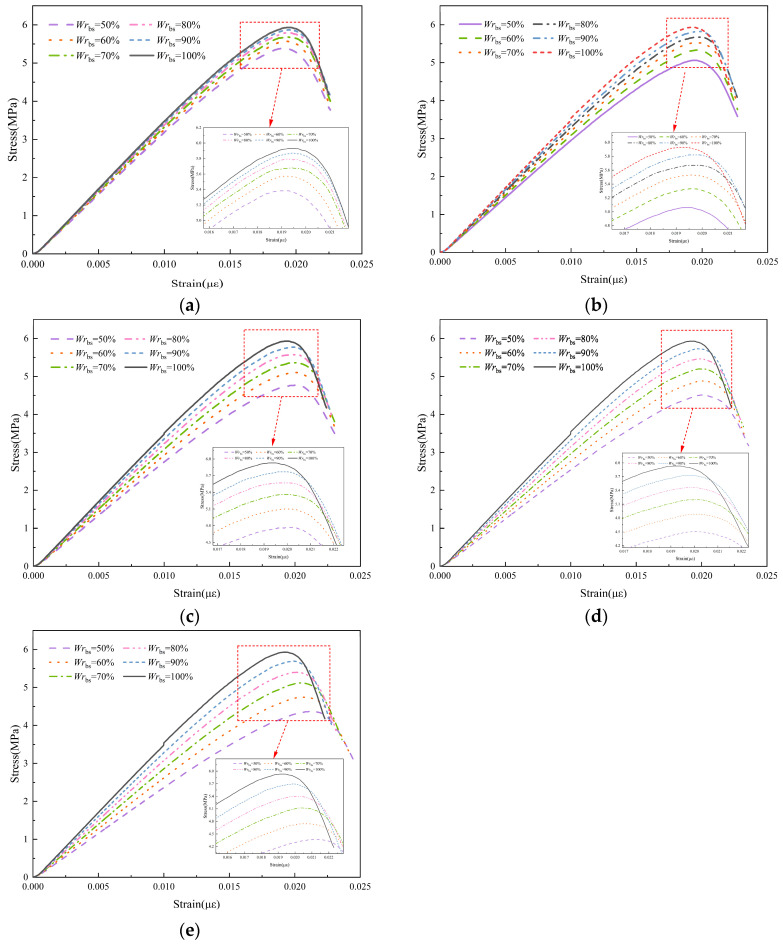
Stress–strain curve of different parameters: (**a**) *Rr_ca_* = 20%; (**b**) *Rr_ca_* = 40%; (**c**) *Rr_ca_* = 60%; (**d**) *Rr_ca_* = 80%; (**e**) *Rr_ca_* = 100%.

The mechanical performance of the weakly bonded CSM is predominantly governed by two key parameters: the replacement ratio of coarse aggregate (*Rr_ca_*) and the weakening ratio of bond strength (*Wr_bs_*). The *Rr_ca_* refers to the proportion of the selected weakened coarse aggregate relative to the total coarse aggregate. The *Wr_bs_* is defined as the ratio of the weakly bonded strength to the normally bonded strength at the coarse aggregate–cement mortar interface. To quantify the performance degradation degree of weakly bonded CSM under various parameter combinations, UCS simulation tests were conducted in this study, as illustrated in Figure 13. The UCS of weakly bonded CSM under various parameter combinations are presented in Table 8.

The test results demonstrate that the UCS of weakly bonded CSM exhibits a distinct parameter response law: its strength decreases with an increase in the *Rr_ca_* and a decrease in the *Wr_bs_*. The maximum strength reduction reaches 26.3% at the extreme condition of *Rr_ca_* = 100% and *Wr_bs_* = 50%. Furthermore, with a decrease in the *Wr_bs_*, the post-peak stress–strain curve of CSM exhibits a progressively gentler slope. This suggests that weak interfacial bonding contributes to improved residual strength retention in the specimens. Consequently, the UCS of weakly bonded CSM under all parameter combinations is consistently lower than that of normally bonded CSM. Nevertheless, within a reasonable matching range of *Rr_ca_* and *Wr_bs_*, the UCS of weakly bonded CSM still meets the strength requirements for pavement base materials specified in China’s current specifications, indicating favorable feasibility for engineering applications.

**Figure 14:**
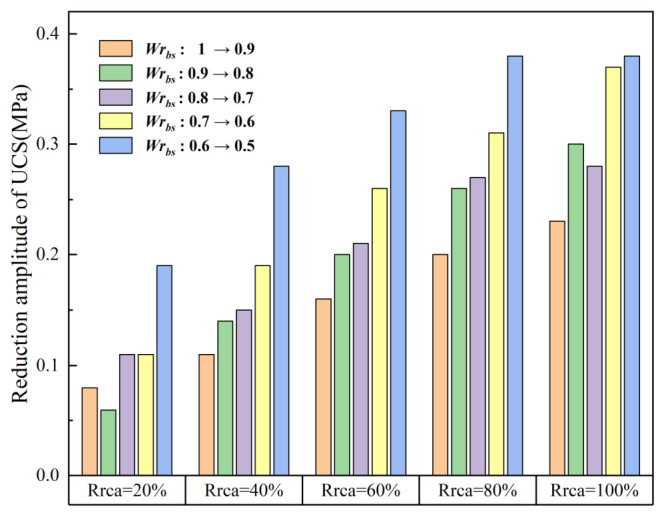
Stress Reduction Under Different Parameters.

Under different *Rr_ca_*, the UCS of weakly bonded CSM decreased with the reduction in the *Wr_bs_*. The reduction amplitude of the UCS was illustrated in Figure 14. The UCS reduction amplitude reached the minimum when the *Wr_bs_* decreased from 1.0 to 0.9; in contrast, the maximum reduction amplitude occurred as the *Wr_bs_* dropped from 0.6 to 0.5. Overall, with the *Wr_bs_* progressively decreasing from 1.0 to 0.5 at a gradient of 0.1, the UCS reduction amplitude exhibited a stepwise increasing trend. A further comparison among groups with different *Rr_ca_* indicated that, under the same *Wr_bs_* conditions, a higher *Rr_ca_* corresponded to a greater reduction amplitude of the UCS of the specimens. Therefore, in practical engineering applications, the *Rr_ca_* should be reasonably selected to ensure that the material strength meets the design requirements.

### 4.2. Prediction Model of UCS

**Figure 15:**
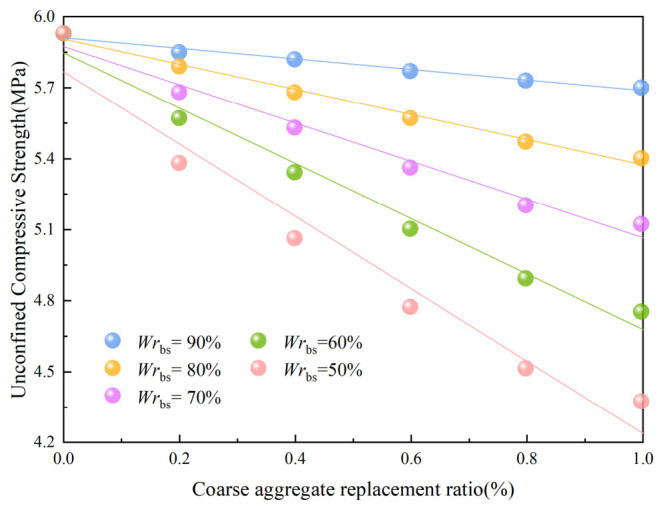
Linear regression results of UCS reduction with different *Wr_bs_*.

In this study, the variation trend diagrams of the UCS of specimens under various *Rr_ca_* were plotted, as shown in Figure 15. A distinct linear correlation is confirmed between the UCS of weakly bonded CSM and *Rr_ca_*. Subsequently, the attenuation formulas of weakly bonded CSM under different *Wr_bs_* were obtained through linear regression analysis, as presented in Table 9.

As observed, the UCS exhibited a consistent decrease with increasing *Rr_ca_* for all levels of the *Wr_bs_*. Linear regression analysis yielded coefficients of determination (R^2^) exceeding 96% for the linear equations corresponding to each bond strength weakening level. This demonstrated a strong linear correlation between the UCS and *Rr_ca_* for weakly bonded CSM with varying interface strengths, under uniform gradation and particle distribution conditions. Nevertheless, the observed correlation is derived from one specific gradation only. Additional verification across diverse gradations, cement contents, and aggregate sources is essential before using it to predict unconfined compressive strength.

## 5. Comparison and Analysis

### 5.1. UCS and Crack Counts

**Figure 16:**
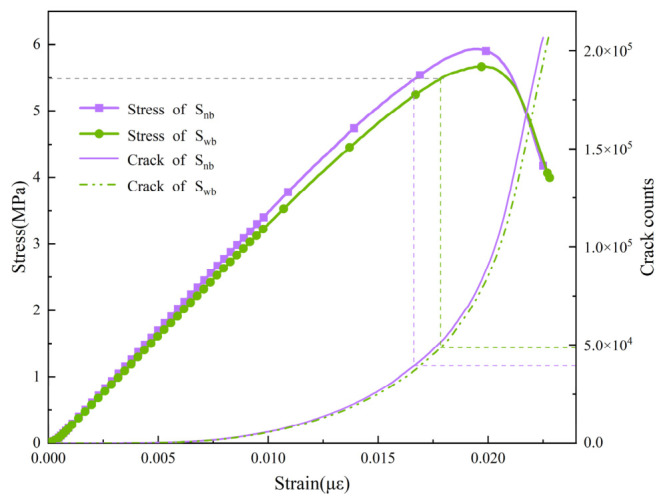
Comparison of UCS and crack number evolution between two bonding types of CSM.

This study aimed to elucidate the mechanism underlying the influence of weakened interface bonding on the failure behavior of cement-stabilized macadam (CSM). Accordingly, specimens with a *Rr_ca_* of 40% and a *Wr_bs_* of 80% (weakly bonded specimens) were selected for comparative analysis with normally bonded specimens. The stress–strain curves and crack evolution curves of the two specimen types are presented in Figure 16.

The results indicated that the UCS of the weakly bonded specimens was 5.68 MPa, approximately 4.2% lower than that of the normally bonded specimens (5.93 MPa). Upon specimen failure, the total number of micro-cracks in the weakly bonded specimens was 209,654, significantly higher than the 207,444 observed in the normally bonded specimens. More notably, the weakly bonded specimens consistently exhibited a higher crack count than the normally bonded ones at the same stress level. This suggested that the weakened interfaces had become the weak links where cracks were more prone to initiate.

### 5.2. Micro-Cracks Distribution of Specimens

**Figure 17:**
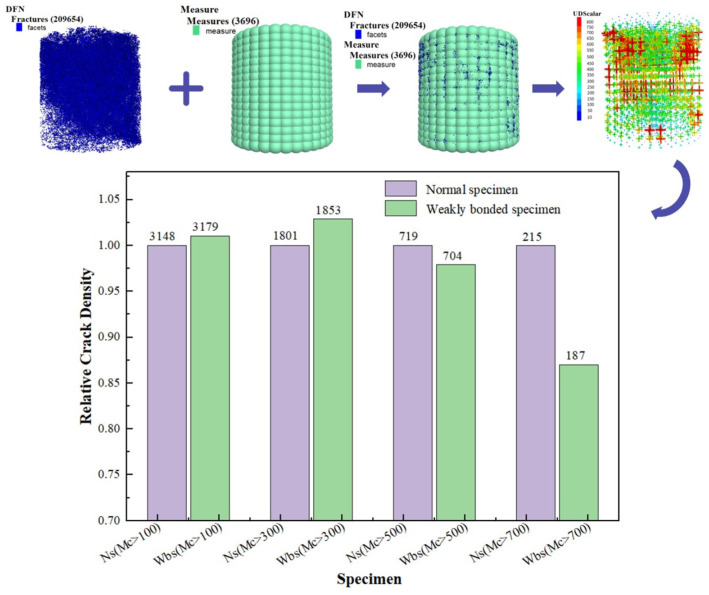
Comparison of crack distribution between two bonding types of CSM.

Using the method described in Section 4.2 of this paper, 3696 regularly arranged measurement spheres were generated in both the weakly bonded and normally bonded specimens. The number of micro-cracks within each sphere was statistically counted, enabling the quantification of the crack aggregation law. The statistical results corresponding to different crack density thresholds were presented in Figure 17.

The data revealed that at lower micro-crack count thresholds (>100, >300), more measurement spheres meeting the criteria were identified in the weakly bonded specimens. Conversely, the normally bonded specimens contained a greater number of qualified measurement spheres at higher thresholds (>500, >700). This indicated that the failure of the normally bonded specimens exhibited a more pronounced localized concentration, with micro-cracks tending to coalesce in specific areas and propagate into dominant penetrating cracks. In contrast, the failure of weakly bonded specimens was characterized by a distinctly global dispersion pattern, with micro-cracks extensively initiating throughout the material and thus inhibiting the formation of large-scale concentrated damage zones.

### 5.3. Energy Evolution Comparison

**Figure 18:**
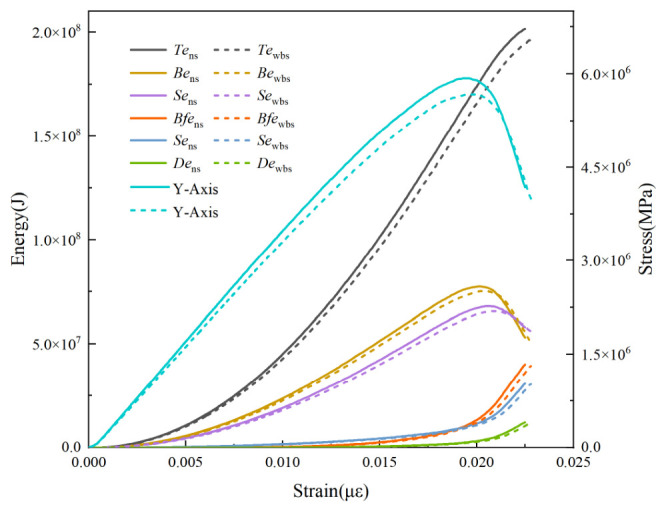
Energy evolution of different specimen: Te is total energy, Be is bonding energy, Se is strain energy, Bfe is bonding failure energy, Se is slip energy, Cn is crack number, ns is normal specimen, wbs is weakly bonded specimen.

In this paper, the evolution of energy in both specimen types during the UCS test was monitored and was presented in Figure 18. The results indicated that the total energy, as well as its individual components, were consistently lower for the weakly bonded specimens compared to the normally bonded ones. This phenomenon is attributed to the reduction in overall stiffness and elastic energy storage capacity induced by interfacial weakening.

Furthermore, the weakly bonded specimens exhibited greater strain values at the peaks of both strain energy and bonding energy, followed by a more gradual post-peak release. Meanwhile, the evolution of their bonding failure energy and frictional energy displayed a more gradual increasing trend. This indicates that the failure process of the weakly bonded specimens transformed from a pattern of concentrated energy release to one of gradual energy dissipation.

We hypothesize that this behavior can be attributed to the following mechanism: the weakened interfaces reduce the crack initiation threshold, promoting the dispersed initiation of more micro-cracks. These micro-cracks prolong the crack propagation path and inhibit the concentrated release of energy along the main crack. Consequently, the material may continue to dissipate energy progressively through sustained interfacial frictional sliding, even after macroscopic strength loss.

## 6. Conclusions

In this study, discrete element models were established for weakly and normally bonded CSM specimens. Virtual unconfined compressive strength (UCS) tests were subsequently conducted, facilitating a systematic comparative analysis of the mechanical strength characteristics, mesoscopic crack evolution laws, and energy dissipation mechanisms between the two types of specimens. Under the simulation conditions adopted in this study, the main research conclusions are as follows:

(1) The failure process of CSM can be divided into four stages: Damage Accumulation Stage, Crack Initiation Stage, Crack Propagation Stage, Penetration, and Failure Stage.
(2) In UCS tests, cracks in CSM specimens are predominantly distributed at the specimen top and within shear failure zones oriented at 45° relative to the axial loading direction.
(3) Although weakly bonded CSM exhibits a reduction in UCS compared to traditional materials, it remains compliant with Chinese specifications when designed with suitable parameters.
(4) Upon loading, the more dispersed distribution of micro-cracks in weakly bonded CSM contributes to suppressing the initiation and propagation of dominant macroscopic cracks.
(5) In contrast to the concentrated energy dissipation of conventional CSM, weakly bonded CSM exhibits a gentler and more sustained energy dissipation behavior, demonstrating a typical ductile energy dissipation mechanism.
(6) The UCS of weakly bonded CSM exhibits an extremely strong linear correlation with the replacement ratio of coarse aggregate (*Rr_ca_*). This finding provides a valuable reference for estimating material strength in practical engineering applications.

This DEM analysis was performed only for a single aggregate gradation and a fixed cement content. The revealed mechanical mechanisms and laws need further verification for generalizability with a wider range of gradations, cement dosages, and aggregate types in future work. Corresponding laboratory tests including fatigue, shrinkage, and freeze–thaw tests will also be supplemented to establish a comprehensive performance evaluation framework.

## Figures and Tables

**Table 1:** Cement technical indexes.

Index	Fineness	Initial Setting Time (min)	Final Setting Time (min)	Stability	Day Strength (MPa)	
	45 μm Sieve Residue (%)				Compression	Anti-Fracture
Result	6.3	245	365	Qualified	48.6	10.4
Requirement	≥5	≥180	≥360		≥42.5	≥6.5

**Table 2:** Technical indexes of coarse aggregate.

Performance Index	Result	Requirement
Crush value (%)	18.8	≤22
Flat elongated particles content (%)	11.5	≤18
Particle content below 0.075 mm (%)	0.5	≤1.2
Soft stone content (%)	1.1	≤3

**Table 3:** Technical indexes of fine aggregate.

Performance Index	Result	Requirement
Size analysis	Qualified	Meet the grading requirements
Plasticity index	12	≤17
Organic content (%)	0.6	≤2
Sulfate content (%)	0.12	≤0.25

**Table 4:** Gradation of the CSM.

**Sieve size** **(mm)**	0.075	0.15	0.3	0.6	1.18	2.36	4.75	9.5	13.2	16	19	26.5
**M** **ass passing** **Rate (%)**	2.4	7.3	10	14.1	20	27.5	36.9	54.9	64.5	76.6	82	100

**Table 5:** Contact models.

Contact Types	Contact Model
Contact between aggregate and cement mortar	Linear Parallel bond model
Contact within cement mortar	Linear Parallel bond model
Contact between aggregate and aggregate	Linear model
Contact between aggregate and wall	Linear model
Contact between cement mortar and wall	Linear model

**Table 6:** Parameter values of the LPBM.

Contact Model	Contact Interface	Emod (GPa)	Kratio	Pb_ten (MPa)	Pb_coh (MPa)	Fric
Linear Parallel Bond Model	Mortar-Aggregate	0.132	1.0	1.3	5.2	0.7
	Mortar-Mortar	0.14	1.0	1.4	5.6	0.7
Linear Model	Aggregate-Facet	1	1.5	-	-	0.5
	Mortar-Facet	1	1.5	-	-	0.5
	Aggregate-Aggregate	0.1	1	-	-	0.7

**Table 7:** Parameter values of the weakly bonded model.

*Wr_bs_*	Emod (GPa)	Kratio	Pb_ten (MPa)	Pb_coh (MPa)	Fric
90%	0.1188	1.0	1.17	4.68	0.7
80%	0.1056	1.0	1.04	4.16	0.7
70%	0.0924	1.0	0.91	3.64	0.7
60%	0.0792	1.0	0.78	3.12	0.7
50%	0.066	1.0	0.65	2.6	0.7

**Table 8:** UCS of different parameters.

*Rr_ca_* (%)	UCS of Specimens Under Different *Wr_bs_* (MPa)					
	Control Group	90%	80%	70%	60%	50%
0	5.93	5.93	5.93	5.93	5.93	5.93
20	5.93	5.85	5.79	5.68	5.57	5.38
40	5.93	5.82	5.68	5.53	5.34	5.06
60	5.93	5.77	5.57	5.36	5.1	4.77
80	5.93	5.73	5.47	5.2	4.89	4.51
100	5.93	5.70	5.4	5.12	4.75	4.37

**Table 9:** UCS reduction rates formulas of specimens with different *Wr_bs_*.

*Wr_bs_* (%)	Linear Fitting Equation	R^2^
90	y=−0.223x+5.911	0.9766
80	y=−0.531x+5.906	0.9904
70	y=−0.809x+5.874	0.9804
60	y=−1.169x+5.848	0.9815
50	y=−1.469x+5.848	0.9606

## Data Availability

The original contributions presented in this study are included in the article. Further inquiries can be directed to the corresponding author.

## Abbreviations

The following abbreviations are used in this manuscript:

CSM	Cement Stabilized Macadam
UCS	Unconfined Compressive Strength
*Wr_bs_*	The weakening ratio of bond strength
*Rr_ca_*	The replacement ratio of coarse aggregate
